# Rhinogenic Optic Neuritis Caused by Sphenoid Mucocele with Sinusitis

**DOI:** 10.1155/2018/8302415

**Published:** 2018-06-20

**Authors:** Sasitorn Siritho, Weerachai Tantinikorn, Paithoon Wichiwaniwate, Krit Pongpirul

**Affiliations:** ^1^Bumrungrad International Hospital, Bangkok 10110, Thailand; ^2^Division of Neurology, Department of Medicine, Siriraj Hospital, Mahidol University, Bangkok 10700, Thailand; ^3^Department of Preventive and Social Medicine, Faculty of Medicine, Chulalongkorn University, Bangkok 10330, Thailand; ^4^Department of International Health, Johns Hopkins Bloomberg School of Public Health, Baltimore, MD 21205, USA

## Abstract

A 59-year-old male who presented with a nonspecific headache at the vertex, resembling retrobulbar optic neuritis, was treated as such but did not show any improvement. Although optic nerve compression from sphenoid mucocele was finally discovered, the delayed diagnosis and improper treatment led to a permanent visual loss. Optic neuritis could be caused by a common problem, “mucocele/sinusitis,” but might be easily overlooked in general practice. Rhinogenic optic neuropathy should, therefore, be considered in every case of optic neuritis whenever atypical presentation occurs or is unresponsive to high-dose steroid treatment.

## 1. Introduction

A 59-year-old healthy male presented with a nonspecific headache at the vertex followed by sudden painless visual loss of the right eye. Ophthalmologic examination showed visual acuity of no light projection with a relative afferent pupillary defect. No diplopia was found. The intraocular pressure, optic disc appearance, and other neurological assessments were normal. Routine blood tests and echocardiogram were unremarkable. Magnetic resonance imaging (MRI) of the brain and orbit performed 2 days after the headache incident revealed minimal fluid surrounding the optic nerve sheath suggestive of mild swelling of the optic nerve sheath papilledema. A follow-up computerized tomography (CT) of the brain 6 days after the onset was unremarkable. With the initial diagnosis of right optic neuritis, he was given intravenous dexamethasone 96 mg/day for 2 days and then switched to oral prednisolone for 3 days.

Because of no improvement within one month, he sought for the second opinion, which revealed persistently no light perception of the right eye with the temporal pallor of the optic disc but normal macula and vascular trunks. Complete blood count, lipid panel, hepatitis profile, anti-HIV, thyroid function, erythrocyte sedimentation rate, C-reactive protein level, autoimmune screening tests, and AQP4-antibody were all unremarkable. Intravenous methylprednisolone 1 g/day for 3 days was given but no improvement was observed.

**Figure 1:**
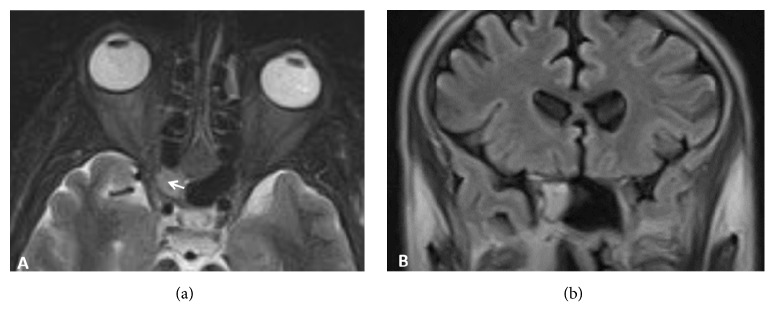
Magnetic resonance images of the brain and orbits. (a) Axial STIR T2W showed questionable mucocele of the right sphenoid sinus or sphenoidal sinusitis (white arrow) but the architecture of the optic nerve was not well defined. (b) Coronal FLAIR T2W showed right sphenoidal pathology but the right optic nerve was not well delineated.

The previous MRI brain and orbit images were reviewed (Figure 1). Axial STIR T2W showed questionable mucocele of the right sphenoid sinus or sphenoidal sinusitis but the architecture of the optic nerve was not well defined. Coronal FLAIR T2W showed right sphenoidal pathology but the right optic nerve was not well delineated. Thinner slice and contrasted study should have been performed for the definite diagnosis of optic neuritis.

Two weeks later, another MRI of the brain and orbit demonstrated focal hyposignal T1/hypersignal T2 retention cyst, adhering to the right lateral sphenoidal wall, around the right optic nerve canal with mild surrounding enhancement, along the mucosa and around the optic nerve canal, and focal mild swelling with ill-defined hypersignal T2 change and faint enhancement of the corresponding intracanalicular portion of the right optic nerve.

**Figure 2:**
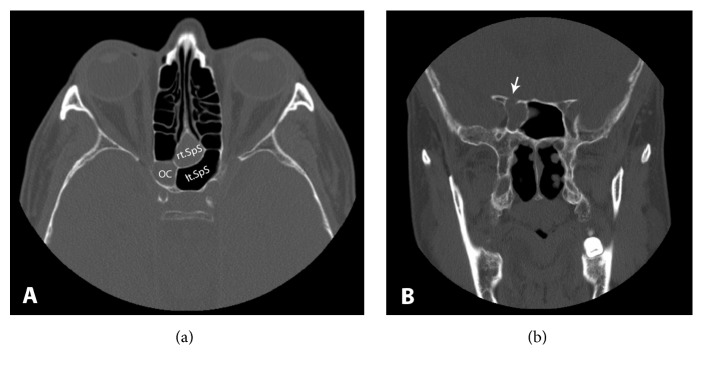
Computerized tomography of paranasal sinus. (a) Anatomical location of Onodi cell (OC); right (rt.SpS) and left sphenoid sinus (lt.SpS). (b) Open roof of superior wall of right sphenoid sinus (white arrow).

The multidetector computed tomography (MDCT) of the paranasal sinuses illustrated a nonenhanced soft tissue density lesion in the right sphenoid sinus and no visualization of the superior wall of the right sphenoid sinus, adjacent to the right optic canal, as well as a wall that became thinner by either pressure effect of right sphenoid lesion or bony destruction (Figure 2). There is a loss of perioptic CSF space at the intracanalicular segment of the right optic nerve. Provisional diagnosis with sinusitis-induced optic neuritis was made.

**Figure 3:**
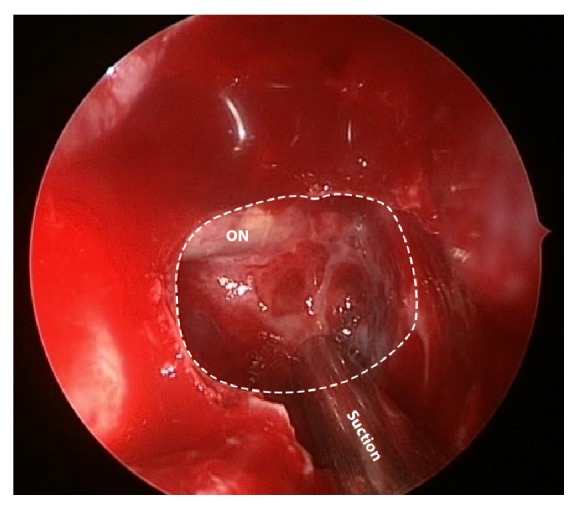
Top view of Onodi cell (white dashed line) showed dehiscence of the bone over the naked right optic nerve.

Right posterior ethmoidectomy, sphenoidotomy, and draining the content in the Onodi cell (Figure 3) were performed and found whitish bloody mucoid discharge in the Onodi cell with dehiscence of the superior wall exposing the right optic nerve. The culture of the pus was negative for organisms. Amoxicillin/clavulanic acid was given for 10 days but the visual acuity did not show further improvement. The patient showed no improvement after the surgery, probably because of the long period of compression as evidenced by the presence of optic atrophy.

## 2. Discussion

The Onodi cell or sphenoethmoidal cell, first described by Adolf Onodi in 1904 [1], is a posterior ethmoid air cell that lies superior to the sphenoid sinus and is in close proximity to the optic nerve or internal carotid artery (ICA) [2–6]. Identification of this structure is useful for minimizing perioperative complication in endoscopic endonasal/transsphenoidal surgery. Because of the anatomical correlation, sinus infection of Onodi cells can lead to inflammation/infection of the optic nerve, so-called "rhinogenic optic neuropathy" [2, 5, 7]. Several mechanisms have been proposed accordingly: (1) direct spreading of the sinus infection through the posterior paranasal sinuses or by osteomyelitis of the sinus wall to the optic nerve [8, 9], (2) compression of the optic nerve caused by ethmoid and/or sphenoid mucoceles [10–14], (3) bacteraemia passing through the mucosa of the sinus [5], (4) vasculitis of the optic nerve [15, 16], and (5) chronic allergic optic neuritis [17].

Our report showed an immunocompetent male patient with atypical unilateral painless retrobulbar neuritis with rapid onset but unresponsive to high-dose steroids. MRI of the orbit and CT of paranasal sinus showed soft tissue density lesion with a very thinning wall of the right sphenoid lesion suggestive of bony destruction of the same side of the suspected ON. The surgical drainage of the right Onodi cell revealed noninfectious discharge; the compression optic neuropathy associated with mucoceles with or without direct spreading from suppurative sinusitis should, therefore, be the pathophysiologic causes.

Two cases were reported from Spain [18]: (1) 21-year-old female presenting with typical unilateral ON and central scotoma without eye pain who was unresponsive to steroid treatment and later developed stuffy nose and nasal symptoms and (2) 75-year old male presenting with rapid onset severe unilateral visual loss, severe scotoma, and papillary pallor who underwent surgical opening of suppurative sphenoidal cyst. The former was clinically improved by antibiotic treatment and the latter received successful surgical correction. Another report was a man who presented with right eye pain on the nasal aspect followed by sudden onset of ON secondary to mucocele and had complete recovery after surgical drainage [19].

Even the rare occurrence of rhinogenic optic neuritis, sinusitis of the Onodi cell causing optic neuropathy, should be considered as a possible cause in all cases of atypical optic neuritis. The recommended imaging to analyze the sphenoid sinus and its surrounding structures is the CT paranasal sinuses including axial, coronal, sagittal, and sagittal oblique (parallel to the optic canal) views. Proper treatments with antibiotics and surgical intervention at the right time are crucial in order to prevent the irreversible optic nerve damage.

## 3. Conclusion

Rhinogenic optic neuritis should be one of the differential diagnoses in patient presenting with atypical neuritis.

## 4. Learning Points

- Optic neuritis could be caused by a common problem “mucocele/sinusitis” but might be easily overlooked in general practice.
- Delayed diagnosis and improper treatment could lead to permanent visual loss.
- The appropriate imaging should be done with fine cut or appropriate sequences if sinusitis of the Onodi cell causing optic neuropathy is suspected.
- Rhinogenic optic neuropathy should be considered in every case of optic neuritis whenever atypical presentation occurs or is unresponsive to high-dose steroid treatment.
